# The role of renal dual-energy computed tomography in exploring the gouty kidney: the RENODECT study

**DOI:** 10.1080/07853890.2025.2458783

**Published:** 2025-01-29

**Authors:** Tristan Pascart, Elie Dauphin, Chio Yokose, Charlotte Jauffret, Aurore Pacaud, Victor Laurent, Vincent Ducoulombier, Hyon K. Choi, Jean-François Budzik

**Affiliations:** ^a^ Department of Rheumatology, Lille Catholic University, Saint-Philibert Hospital, ETHICS laboratory, Lille, EA, France; ^b^ Massachusetts General Hospital, Harvard Medical School, Boston, MA, USA; ^c^ Department of Medical Imaging, Lille Catholic University, Saint-Philibert Hospital, ETHICS laboratory, Lille, EA, France

**Keywords:** Gout, chronic kidney disease, dual-energy computed tomography, monosodium urate, uric acid

## Abstract

**Objective:**

The objective of this study was to explore the ability of dual-energy computed tomography (DECT) to detect monosodium urate (MSU) crystal deposits in the kidneys and renal artery walls, and uric acid urolithiasis, in patients with gout and chronic kidney disease (CKD).

**Methods:**

Patients with gout and with stage 2–4 CKD were prospectively included in this cross-sectional study. Patients underwent renal, knee and feet DECT scans. Renal DECT scans were read for MSU-coded lesions in the kidneys, renal artery walls, and urinary tract using different post-processing settings. Characteristics of patients with and without DECT-positive lesions were compared, and the DECT parameters of these lesions were measured.

**Results:**

A total of 27/31 patients with had renal DECT scans and were included in the analysis (23/27 men, mean (standard deviation) 73 (9) years old, mean eGFR 45.3 mL/min/1.73 m2 (21.0), volumes of MSU in the knees and feet ranging from 0.11 to 475.0 cm^3^). None of the patients exhibited deposition of MSU crystals in the kidneys. One case of calyceal calculi and one case of ureterolithiasis were observed, wrongly coded as MSU in default post-processing settings for gout but identified as uric acid in the “kidney stone” settings. Five patients had MSU-coded plaques in the renal arteries, which had DECT parameters consistent with early calcified plaques rather than MSU, and had no association with volumes of peripheral MSU deposition.

**Conclusion:**

DECT is unable to detect genuine monosodium urate crystal deposits in kidneys and renal artery walls, and but can characterize chronic asymptomatic urolithiasis.

## Introduction

Gout is the most common cause of inflammatory arthritis worldwide [1]. Prolonged hyperuricemia beyond the saturation level of urate leads to the crystallization of monosodium urate (MSU) which deposits inside joints and soft tissue [1]. Once deposited, MSU crystals can induce acute and chronic local and systemic inflammation, particularly inside joints, causing excruciatingly painful gout flares.

Chronic kidney disease (CKD) is a common feature in patients with gout, and gout is a common complication of CKD, creating many management issues [2]. CKD can occur in gout due to multiple suspected or established causes, some of which are gout-specific [3]. Gout nephropathy, a debated entity for decades, refers to the deposition of MSU crystals inside the renal medulla or cortex which have been evidenced by histological examination and suggested by ultrasound, and is suspected to contribute to CKD through persistent crystal inflammation in the renal tissue [3,4]. Hyperuricemia is associated with chronic uric acid urolithiasis, which can lead to CKD [5]. Vascular nephropathy is common but not gout-specific, as it is more directly explained by comorbidities associated with gout [6,7]. There is however an ongoing debate on whether MSU crystals could deposit in artery walls and explain the increased risk of cardiovascular events in gout [8]. Note that the crystal form of uric acid in the body is urate (MSU), as the normal pH in tissues (and blood) is alkaline, contrary to urine in which uric acid crystals can form.

Dual-energy CT (DECT) uses two X-ray beams of different energies (low and high) to characterize body structures and lesions. The underlying principle of the technique in its application to identify and characterize crystal deposits is that the X-ray beam attenuation (i.e. tissue absorption) in a tissue containing a sufficient quantity of crystals (in the case of gout MSU crystals), unlike the same crystal-free tissue, varies with the level of the X-ray energy (usually 80 and 140 kV) [9]. DECT has been extensively demonstrated to identify MSU crystal deposition around joints [9] but its ability to detect MSU crystals in the kidneys and vessel walls is unclear [10–13]. Optimizing DECT post-processing default settings improves the specificity of MSU deposit detection [14]. The ability of DECT to effectively characterize the chemistry of kidney stones, and in particular to distinguish uric from non-uric stones, has been well demonstrated [15]. The objective of this study was to explore whether DECT can detect MSU crystal deposits in the renal medulla or cortex and renal artery walls, and uric acid urolithiasis, in patients with gout and CKD.

## Methods

### Patients

Patients from the CRYSTALILLE cohort with a diagnosis of gout according to the 2015 ACR/EULAR classification criteria and with stage 2–4 CKD were prospectively included in this exploratory cross-sectional study [16,17]. The study was approved by the French ethics committee CPP Sud-Est III (EudraCT 2020-A01269-30) (approval for the CRYSTALILLE cohort), and patients provided oral informed consent recorded in their medical records. The study adhered to the Declaration of Helsinki. Patients’ demographics, comorbidities, gout characteristics and treatment, current serum urate (SU) levels and estimated glomerular filtration rates (eGFR) were recorded.

### DECT protocol

DECT scans were performed using a dual-source CT system (Somatom Drive; Siemens Healthineers, Erlangen, Germany). The abdominal region of the kidneys was scanned with patients positioned supine using the following acquisition parameters: tube potentials, 100 and 140 kV; quality reference tube current-time and maximal products at 100 and 140 kV, 627–798 and 800 mAs, respectively; beam collimation 128 × 0.6 mm; overall volume CT dose indexes, 20–25 mGy. Data for the knees and feet were acquired with patients positioned supine using the following parameters: tube potentials, 80 and 140 kV; quality reference tube current-time and maximal products at 80 and 140 kV, 331–386 and 525 mAs, respectively; beam collimation, 128 × 0.6 mm; overall volume CT dose indexes, 5.6–7.2 mGy.

### Image analysis

DECT images were post-processed and analyzed using syngo.*via* VB10B (Siemens Healthineers). With the “gout” setting, any areas meeting the attenuation properties of MSU are color-coded in green, whereas with the “kidney stones” setting, any areas meeting the attenuation properties of uric acid are color-coded in red. For the knees and feet, the default “gout” settings mode was used to measure volumes of MSU crystal deposition after carefully identifying artifacts including submillimetric lesions. Abdominal scans were processed in “gout” mode, first using default settings (iodine ratio 1.22) then “optimized” settings (iodine ratio 1.09) to increase the specificity of the settings [14]. Images were examined for green MSU-coded lesions in both modes in the renal artery walls, kidney medulla or cortex and urinary tract. Kidney scans were then analyzed using the “kidney stones” mode, and images were examined for red uric acid-coded lesions. Only lesions >2 mm in diameter were considered to avoid artifacts. Regions of interest (ROIs) were manually drawn to encompass each lesion noted on the kidney scans in any mode, and on 4 tophi for reference. Using the “Rho/Z” mode, the following values were provided for each ROI: CT numbers (in HU) at low (80 or 100 kV) and high (140 kV) tube potentials, electron density (Rho), and effective atomic number (Zeff). The interaction between MSU and X-ray relies on Compton scattering which predominates at high energies and is related to the tissue volumetric mass and provides an assessment of the electron density (Rho) of a considered voxel, which increases in a tissue containing sufficient quantities of MSU crystals [9]. The Zeff provides the mean atomic number of the considered volume, and its assessment reflects the photoelectric effect of the tissue. Typically, monosodium urate deposits have a low atomic number and their presence has a neutral effect on the photoelectric effect while increasing the tissue volumetric mass, while biological materials with higher atomic number values such as calcium (calcium pyrophosphate, basic calcium phosphate, bone) generate an increased photoelectric effect. Uric acid crystals found in the urine have an even lower Zeff than MSU crystals [9].

### Statistical analysis

Qualitative variables were described as numbers and percentages, and quantitative variables as means (SD) or medians [Q1–Q3 interquartile range]. Characteristics of patients with and without lesions coded as MSU or uric acid in the renal tissue, renal artery walls and urinary tract were described. Biochemical characteristics of the lesions (CT numbers at low and high energies, Rho and Zeff) were described according to location and positivity in the different post-processing modes. The combined attenuations at low and high energies were reported on a graph, and the position of the dots of each ROI was examined for their distance from the isoattenuation line. It was decided to include an arbitrary number of 30 patients in this exploratory study.

## Results

### Clinical characteristics of patients with positive MSU or uric acid-coded lesions

**Figure 1.**
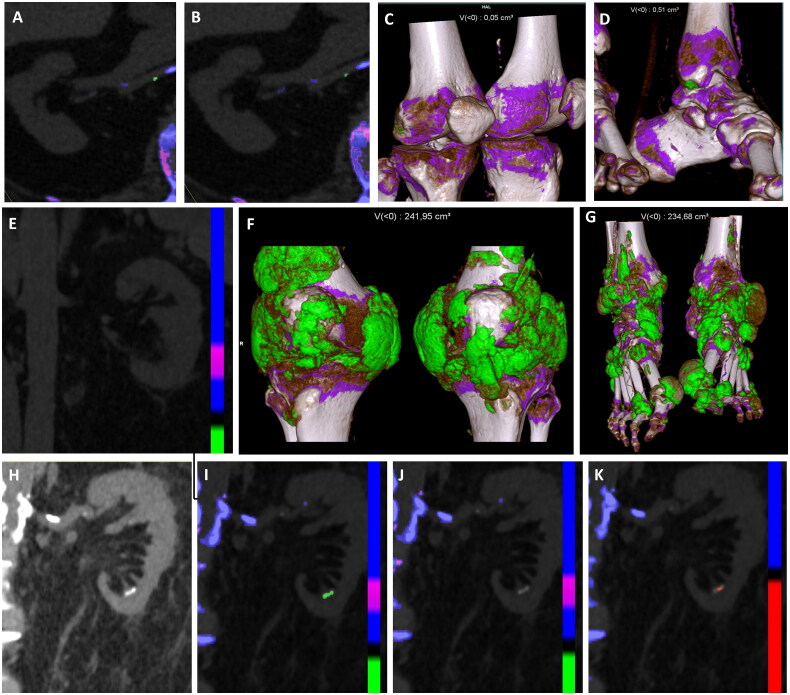
Presence of monosodium urate (MSU)-coded plaque in the right renal artery in default “gout” (2.5 mm) (a), and submillimetric in optimized (b) settings in a 60-year-old patient with moderate MSU deposits in the knees (c) and feet (d). Absence of any renal or vascular MSU deposits (e) in a 61-year-old patient with extensive MSU crystal deposits in the knees (f) and feet (g). Presence of calyceal calculi in (H) native conventional CT images, coded as monosodium urate (MSU) crystals in default “gout” settings (i), uncoded in optimized “gout” settings (j), and coded as uric acid in “kidney stones” settings (k).

A total of 27/31 patients underwent renal DECT scans and were included in the analysis (23/27 (85.2%) men, mean (SD) 73 (9) years old). Gout duration was 5 [2;7] years and current SU levels were 9.2 (3.1) mg/dL (Table 1). A total of 19/27 (70.4%) patients had knee and feet DECT scans positive for MSU crystal deposition (cumulative volumes ranging from 0.11 to 475.0 cm^3^). Mean eGFR was 45.3 mL/min/1.73 m^2^ (21.0), and renal artery calcifications were observed in 20/27 (74.1%) patients. None of the patients exhibited MSU crystal deposition in the renal tissue (no evidence of gout nephropathy) (Figure 1(A–G)). Three participants had urolithiasis. One case of calyceal calculi and one case of urinary tract lithiasis both coded as MSU crystals in default “gout” settings, “disappeared” (or became submillimetric) with optimized “gout” settings, and were coded as uric acid in the “kidney stone” settings (Figure 1(H–K)). One patient presenting with urinary tract lithiasis appeared as MSU-positive in default “gout” settings, was submillimetric in optimized settings, and was not coded as uric acid in “kidney stones” settings. Five patients had MSU-coded plaques both in default and optimized “gout” settings in renal arteries. Participants with DECT arterial MSU-coded plaques were younger than those with kidney DECT scans negative for MSU or uric acid (64 vs 77 years), had similar SU levels (8.8 mg/dL vs 8.9 (2.9)), a higher eGFR (56 ml/min/1.73 m^2^ vs 36), and had smaller volumes of MSU deposits in the knees and feet (0.50 cm^3^ vs 2.48). MSU-coded plaques were systematically associated with renal artery calcifications. The characteristics of patients with urolithiasis did not differ from the other participants.

**Table 1.** Characteristics of participants with negative kidney dual-energy CT (DECT) scans for monosodium urate (MSU) or uric acid deposition, with MSU-coded renal artery plaques and DECT-positive urolithiasis. In the group of patients without any renal lesions, DECT parameters were measured in tophi, which provided typical values expected for MSU crystal deposits.

	Patients with negative kidney DECT (*n* = 20)	Patients with MSU-coded renal artery plaques (*n* = 5*)	Patients with MSU + and uric acid + urolithiasis (*n* = 2*)	Patients with MSU + and uric acid - urolithiasis (*n* = 1)
Age (years) (median [Q1–Q3])	77 [70–81]	64 [62–71]	62–74	58
Men (*n* (%))	17 (85)	5 (100)	2 (100)	1 (100)
Gout duration (years) (median [Q1–Q3])	5 [2–7]	5 [4–20]	20–2	4
Ongoing urate lowering therapy (*n* (%))	6 (30)	3 (67)	0 (0)	1 (100)
Serum urate level (mg/dL) (median [Q1–Q3])	8.9 [7.0–10.6]	8.8 [8.8–13.0]	8.8–10.2	8.8
eGFR (mL/min/1.73m^2^)(median [Q1–Q3])	36 [29–53]	56 [55–74]	78 − 35	74
*DECT results*				
DECT positive for MSU crystal deposition in knees and feet (*n* (%))	17 (85)	4 (100)	1 (50)	1 (100)
DECT volume of MSU crystals in knees and feet if positive (cm^3^)	2.48 [0.27–5.29]	0.50 [0.46–0.73]	0.50	0.46
Vascular calcifications (*n* (%))	13 (65)	5 (100)	2 (100)	1 (100)
Low energy attenuation (HU)(median [Q1–Q3])	193 [183–203]^#^	226 [205–238]	201 − 240	263
High energy attenuation (HU) (median [Q1–Q3])	199 [186–208]^#^	197 [179–206]	227 − 254	250
Electronic density Rho (median [Q1–Q3])	198 [178–202]^#^	208 [185–210]	219 − 216	210
Effective atomic number Zeff (median [Q1–Q3])	7.45 [7.39–7.52]^#^	8.00 [7.83–8.07]	6.58 − 8.00	8.60

DECT: dual-energy computed tomography; Q: quartile; MSU: monosodium urate.

* One patient had both lesions.

^#^ DECT parameters were measured in knee and feettophi.

### Characterization of MSU or uric acid-coded lesions

**Figure 2.**
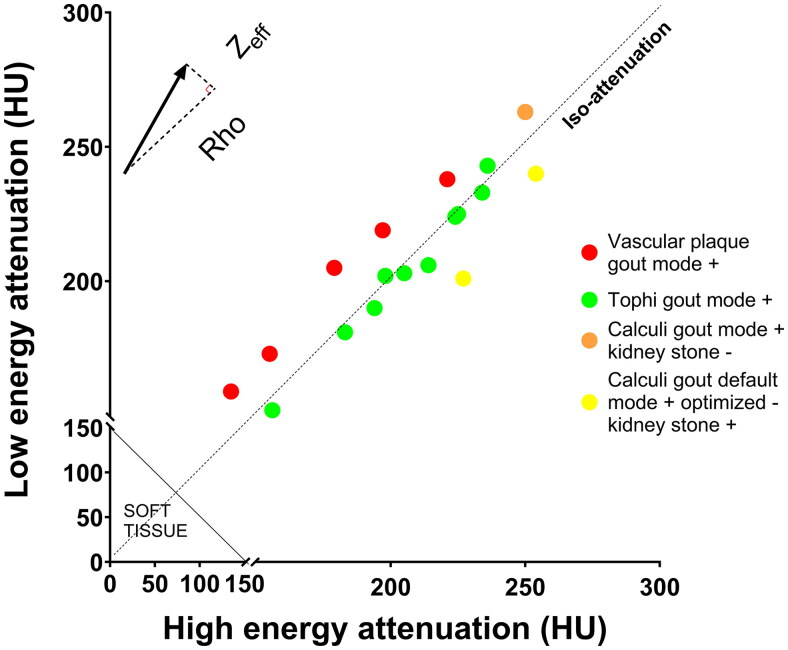
Combined effects of volumetric mass density (Rho) and effective atomic number (Zeff) on CT numbers at 80 and 140 kV on tophi, arterial plaques and urinary tract deposits. Each dot representing a combined value of attenuations at 140 and 80 kV will be coded as MSU (green) if above 150 HU and loosely (default settings) or closely (optimized settings) around the line where attenuations at both energies are equal (isoattenuation). Dots below the isoattenuation line will be coded as uric acid in “kidney stones” mode, while those above the line (higher Zeff) will be coded as containing calcium if in sufficient concentration. Typical MSU deposits (tophi) are distributed closely to the isoattenuation line. MSU: monosodium urate, HU: Hounsfield unit.

Each type of lesion (vascular MSU-coded plaques, urinary tract deposits coded in default/optimized “gout” and “kidney stones” settings, tophi) exhibited specific DECT parameter values (Table 1 and Figure 1). Rho and Zeff values of MSU-coded vascular plaques were different from those observed in tophi and suggested that they are early calcified plaques, particularly because they exhibited higher Zeff values than expected for MSU and just below the predefined threshold to be coded as containing significant quantities of calcium (Figure 2). Characterization of urolithiasis positively coded in “gout” settings but not in “kidney stones” settings suggesting that the kidney stones contained calcium. The urolithiasis cases positive for uric acid were also positive in default “gout” settings, despite a lower attenuation at low energy than at high energy, which is characteristic of uric acid rather than MSU.

## Discussion

This study shows that despite promising abilities, DECT was not able to detect evidence of gout nephropathy, even in patients with extensive MSU crystal deposition around the joints which were expected to be at risk of also presenting deposits in the renal tissue. Caution is warranted when DECT identifies MSU-coded plaques in renal arteries, unrelated to the general MSU crystal burden, which are most probably miscoded due to calcified plaques. As expected, kidney DECT scans were able to detect and characterize uric acid urolithiasis in people with gout and CKD.

Optimizing the post-processing settings is essential to provide the most sensitive and specific results for MSU or uric acid detection. It is now common knowledge when using DECT to detect MSU crystal deposition around peripheral joints that avoiding artifacts is essential to accurately identify and quantify these deposits [9,18]. Using optimized settings in “gout” mode and eliminating submillimetric lesions, retaining only lesions of clinical significance, is determinant in avoiding artifacts [14,19]. Our study shows that using default settings on kidney DECT scans is too permissive and inaccurately classifies uric acid kidney stones as MSU, while optimized settings are sufficiently specific. The applies to vascular plaques coded as MSU in default settings, most often becoming <2 mm with optimized settings, and exhibiting DECT parameters consistent with early calcification, suggesting that these vascular lesions are not MSU [11]. MSU-coded plaques of coronary arteries and the thoracic aorta had been found in 86% of participants with gout (using gout default settings) in a prior study, and in 25% of popliteal arteries in a study from our team, both above the 18.5% of the current study [10,11]. In both cases, these MSU-coded plaques were associated with vascular calcifications, which were less common in renal arteries in the current study. All renal MSU-coded plaques were also associated with the presence of renal artery calcifications, further supporting the artefactual nature of this MSU-coding [8].

Despite significant and sometimes massive MSU crystal deposition around the joints in ULT-naïve patients with high SU levels, no evidence of MSU deposition in the renal medulla (gout nephropathy) was observed. There has been 1 case report of a patient with Lesch-Nyhan syndrome and another with tophaceous gout in whom kidney DECT demonstrated extensive MSU-coded deposition in the renal medulla, with ultrasound showing hyperechoic lesions [12]. The reason for the discrepancy between this report and our study is unclear, and may lie in the specifics of the two previously reported patients, or in the post-processing settings used in that previous report, as the DECT-evidenced MSU deposits were not explored by histopathology, nor were the DECT parameters of the lesions reported. However, none of our patients underwent kidney ultrasound, and our assessment of the risk of gout nephropathy relied on the assessment of the peripheral MSU burden.

Our study has inherent limitations. First, the debated entity of gout nephropathy, if it exists, has a rare occurrence, suspected to affect around 10% of patients with gout [4]. Despite an effort to include participants with a large range of MSU burden, given the small size of this exploratory study and in average only moderate CKD, it is possible that none of the patients included in our exploratory sample were affected with gout nephropathy. The second limitation is technical, as DECT has a lower spatial resolution in abdominal scans than in peripheral joints, and substantial MSU deposits are needed to be detectable in kidneys. Yet, they had been detected in the two case reports. Finally, we did not perform reliability analyses of the measurements of DECT parameters in DECT-positive lesions, but similar measurements have previously been performed on popliteal arteries and provided consistent results [11].

In conclusion, DECT can detect asymptomatic uric acid urolithiasis both in the urinary tract and in the calyces in patients with gout, positively coded in “kidney stones” settings, but miscoded as MSU in default “gout” settings. DECT scans do not appear to be capable of detecting MSU deposits in the renal tissue or in the vessel walls.

## Data Availability

Summary statistics can be requested by researchers for use in independent scientific research and will be provided following review and approval of the research proposal (including the statistical analysis plan) and completion of a data sharing agreement with the Lille Catholic University Hospitals. Requests should be sent to the corresponding author.
